# Clinical and Laboratory Characteristics of Normal Weight and Obese Individuals with Non-Alcoholic Fatty Liver Disease

**DOI:** 10.3390/diagnostics12040801

**Published:** 2022-03-24

**Authors:** Anca Trifan, Adrian Rotaru, Remus Stafie, Ermina Stratina, Sebastian Zenovia, Robert Nastasa, Laura Huiban, Tudor Cuciureanu, Cristina Muzîca, Stefan Chiriac, Irina Gîrleanu, Ana-Maria Sîngeap, Catalin Sfarti, Camelia Cojocariu, Carol Stanciu

**Affiliations:** 1 Department of Gastroenterology, Grigore T. Popa University of Medicine and Pharmacy, 700115 Iasi, Romania; ancatrifan@yahoo.com (A.T.); stratina.ermina@yahoo.com (E.S.); sebastianzenovia20@gmail.com (S.Z.); robertnastasa948@gmail.com (R.N.); huiban.laura@yahoo.com (L.H.); drcuciureanutudor@gmail.com (T.C.); lungu.christina@yahoo.com (C.M.); stefannchiriac@yahoo.com (S.C.); gilda_iri25@yahoo.com (I.G.); anamaria.singeap@yahoo.com (A.-M.S.); cvsfarti@gmail.com (C.S.); cameliacojocariu@yahoo.com (C.C.); stanciucarol@yahoo.com (C.S.); 2 Institute of Gastroenterology and Hepatology, “St. Spiridon” University Hospital, 700111 Iasi, Romania

**Keywords:** liver stiffness, liver steatosis, lean, obese, VCTE

## Abstract

Non-alcoholic fatty liver disease (NAFLD) has had, over the past few decades, a progressively growing prevalence among the general population all over the world, in parallel with metabolic conditions such as type 2 diabetes mellitus (T2DM), dyslipidemia, and obesity. However, NAFLD is also detected in 10–13% of subjects with a body mass index (BMI) ≤ 25 kg/m² (lean-NAFLD), whose major risk factors remain unknown. In this study, we aimed to characterize the clinical features and associated risk factors of lean-NAFLD in comparison with obese-NAFLD patients. Consecutive patients diagnosed with NAFLD by vibration-controlled transient elastography and controlled attenuation parameter were prospectively enrolled. Biological and clinical data obtained from the participants were stratified according to their BMI in two groups: lean-NAFLD and obese-NAFLD. In total, 331 patients (56.8% males) were included in the final analysis. Most of the subjects were obese-NAFLD (*n* = 258, 77.9%) and had a higher prevalence of T2DM, dyslipidemia, and components of the metabolic syndrome, together with abnormal biological parameters. Regarding liver stiffness measurements, the proportion of subjects with at least significant fibrosis (≥F2) was approximately twofold higher among obese-NAFLD (43.81%) in comparison with lean-NAFLD patients (23.29%). Moreover, obese individuals had a higher risk for liver fibrosis (OR = 2.6, 95%, CI 1.5–4.42, *p* < 0.001) than lean individuals. Although associated metabolic conditions and at least significant liver fibrosis were present in approximately one-quarter of the patients, these were more frequent among obese-NAFLD patients. Therefore, individualized screening strategies for NAFLD should be established according to BMI.

## 1. Introduction

Non-alcoholic fatty liver disease (NAFLD) is the most common chronic liver disease globally, with a prevalence varying according to diagnostic methods, ethnic groups, and the evaluated region [1]. Generally, NAFLD affects around a quarter of the general population worldwide and is defined by the presence of excessive fat in more than 5% of the hepatocytes in patients without any history of significant alcohol consumption (<30 g/day in men and <20 g/day in women) or other secondary causes of liver steatosis or fibrosis (e.g., drug-induced liver injury, autoimmune hepatitis, hepatic virus B or C chronic infection) [2,3,4,5]. The term NAFLD refers to a spectrum of different hepatic alterations, from simple steatosis to non-alcoholic steatohepatitis (NASH), with a high risk of progression to end-stage liver disease—namely, liver cirrhosis and hepatocellular carcinoma (HCC) [6,7,8].

NAFLD is strongly linked to the obesity pandemic and overweight status; however, this disorder is also present in lean subjects with body mass index (BMI) < 25 kg/m², with no known evident risk factors [9,10]. The term of lean-NAFLD was first introduced by Vos et al. in 2011, and since then, the prevalence of this disorder seems to be much higher than it was previously believed, mainly due to the availability and accessibility of new non-invasive diagnostic methods in recent times; thus, an early diagnosis is essential for more effective management [7,11,12].

Lean-NAFLD is considered to be a major clinical and diagnosis challenge because when obesity, which is known as a clinical landmark for steatosis, is absent, the diagnosis of liver steatosis or liver damage is oftentimes delayed or even failed to be noticed at all. As a result, the required medical intervention and treatment are most likely initiated too late. In the diagnosis process, helpful hints could be provided by the fact that lean-NAFLD and overweight/obese-NAFLD patients do share a common metabolic profile, closely associated with the components of the metabolic syndrome (MeS), such as hypertension, low high-density lipoprotein level (HDL-c), hypertriglyceridemia, type 2 diabetes mellitus (T2DM) or increased fasting plasma glucose and increased waist circumference [7,13,14].

The pathophysiology of NAFLD is yet to be completely understood, especially in individuals with normal BMI. Even in the presence of normal subcutaneous fat and a low BMI, abdominal fat or central obesity alongside insulin resistance could play an important role in the development of NAFLD in lean subjects [15]. Consequently, body composition measurements, particularly body fat percentage (BF%), could provide additional helpful information in order to better understand this disease [16]. The most accurate methods for determining BF% are magnetic resonance imaging (MRI), computed tomography (CT) scans, and dual-energy X-ray absorptiometry (DXA) [17].

The gold standard method for the diagnosis and staging of the severity of the disease in patients with NAFLD is still considered to be the liver biopsy (LB). However, due to its invasive nature and possible complications, this method cannot be used as a screening option, giving way to an increasing number of non-invasive cost-efficient methods, such as biochemical tests or imaging techniques [18,19]. Among them, vibration-controlled transient elastography (VCTE) with controlled attenuation parameter (CAP) is considered to be the non-invasive method of choice, allowing simultaneous assessment of both hepatic steatosis and fibrosis, with high rates of sensitivity and specificity for staging chronic liver diseases and a low failure rate (less than 5% since the XL-probe was introduced in the machine equipment) [20,21].

Therefore, this paper aims to characterize the clinical features and associated risk factors of lean-NAFLD in comparison with obese-NAFLD patients.

## 2. Materials and Methods

### 2.1. Participants

This is a prospective study from a tertiary referral hospital in northeastern Romania that included consecutively lean and obese patients with clinically suspected (elevated liver enzymes) or diagnosed NAFLD by abdominal ultrasonography (US) who were referred to our clinic by general practitioners and colleagues in other specialties; their data were evaluated between November 2019 and October 2021. Demographic data, anthropometric measurements, clinical examination, personal medical history, and VCTE with CAP assessment were recorded for all patients. The inclusion criteria were (1) adults over 18 years, (2) without any history of significant alcohol consumption (<20 g/day in women, <30 g/day in men), (3) no known history of malignancy diagnosed in the past year, (4) without any secondary causes of chronic liver disease, and (5) patients with reliable transient elastography examination (>10 valid measurements with an interquartile range/median (IQR/M) ratio < 30%). Blood tests were collected (anti-HCV Ab, HBsAg, total cholesterol, low-density lipoprotein cholesterol (LDL-c), HDL-c, triglycerides, total bilirubin, gamma-glutamyl transpeptidase (GGT), alanine and aspartate aminotransferase (ALT, AST), fasting plasma glucose, platelets count, serum uric acid (SUA), serum urea, creatinine, C-reactive protein (CRP), alkaline phosphatase (ALP), international normalized ratio (INR), albumin). With regard to their BMI, patients were divided into two sub-groups—namely, lean and obese cohorts. This study was approved by the Ethics Committee of our University and was conducted according to the principles of the Declaration of Helsinki. Each participant signed written informed consent. 

### 2.2. Liver Stiffness Measurements (LSM) and CAP Assessment 

The examinations were performed by two experienced physicians using FibroScan^®^ 502 Touch (EchoSens, Paris, France) following procedure instructions [22]. The examination was performed starting with the M probe on the right hepatic lobe through the 9th to 11th intercostal spaces on the midaxillary line after overnight fasting, with the participant lying in the dorsal decubitus position and his right arm in maximal abduction, while the XL probe was used according to the machine instructions in obese patients. LSM was expressed in kilopascals (kPa) with the following cut-off values for liver fibrosis: ≥5.6 kPa—mild (F1), ≥7.2 kPa—significant (F2), ≥9.5 kPa—advanced (F3), and ≥12.5 kPa—liver cirrhosis (F4) [23]. In addition, for liver steatosis, the results were measured in decibels/meter (dB/m) and the cut-off values were 248 dB/m, 268 dB/m, and 280 dB/m, respectively, for mild (S1), moderate (S2), and severe steatosis (S3) [24]. 

### 2.3. Anthropometric Measurements 

Anthropometric indexes, such as height and weight, were recorded for every patient in optimal conditions (no shoes and light clothes). Cut-off values for BMI established by World Health Organization were used in order to define the two study groups (lean and obese subjects) [25]. 

### 2.4. Statistical Analysis

Descriptive statistics were computed for all factors using IBM SPSS, Version 22.0 (IBM SPSS Inc. Chicago, IL, USA). We used the Kolmogorov–Smirnov test for checking the normality of the distribution of numerical variables. Continuous variables are expressed as means and standard deviation, while categorical ones are expressed as numbers (percentage). Data were analyzed using unpaired *t*-test for comparison of continuous variables between groups for normally distributed data or chi-squared and Fisher’s exact test depending on data type, while Mann–Whitney U test or the Kruskal–Wallis analysis of variance (ANOVA) test was used to compared skewed data as appropriate. Univariate linear regression, followed by multivariate linear regression using only the significant factors, was performed to identify the factors that have a high influence on the CAP and LSM values. Pearson correlation coefficient (r) was used for establishing the association between two variables. A two-tailed *p*-value of <0.05 was considered statistically significant. Only complete datasets were analyzed.

## 3. Results

### 3.1. Patient Characteristics

**Figure 1:**
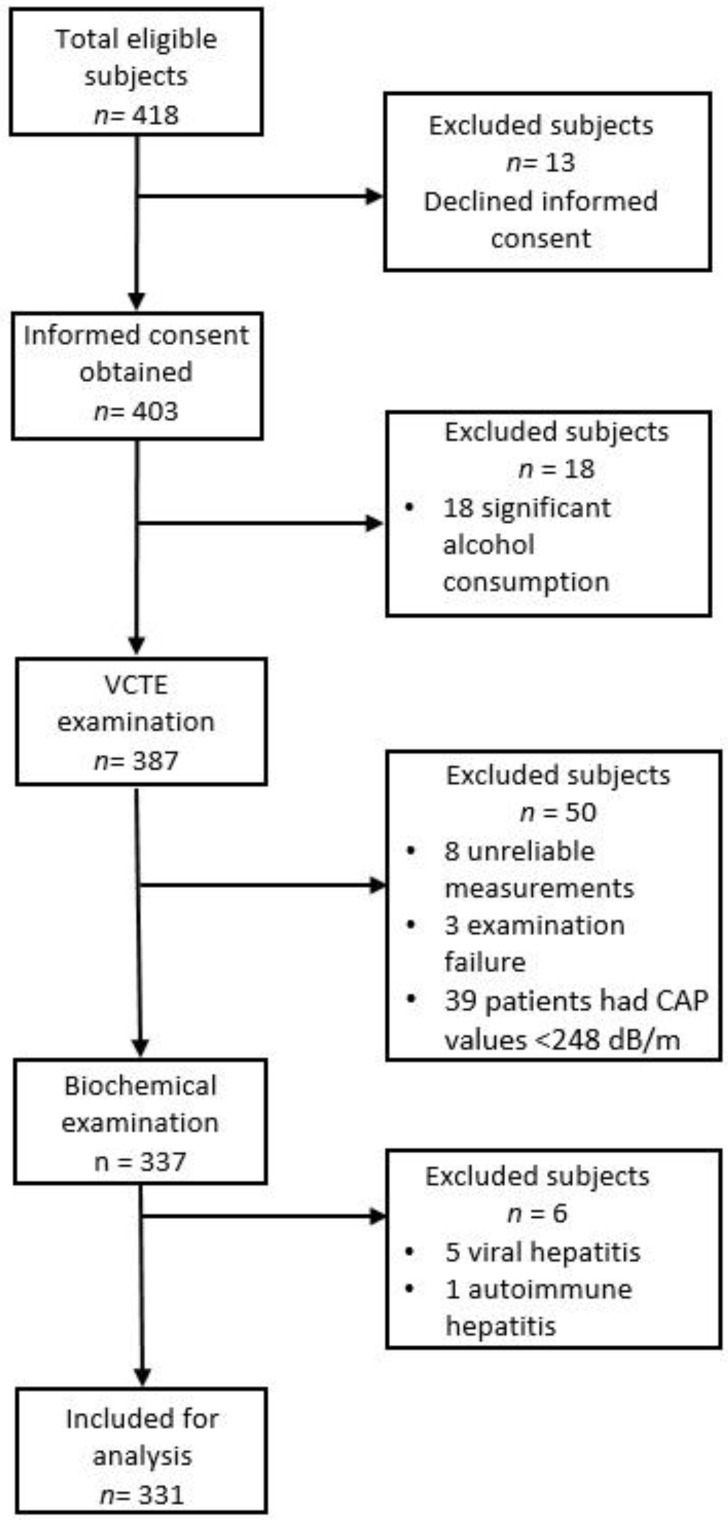
Participant flowchart.

In our study, 418 lean and obese patients with clinically suspected or diagnosed NAFLD were evaluated (Figure 1). 

In total, 13 subjects declined the informed consent, while 18 patients were excluded because of a history of significant alcohol consumption. A total of 387 patients were examined using VCTE with CAP, 50 subjects being excluded afterward (8 subjects with unreliable measurements, 3 individuals with examination failure, and 39 patients with CAP values < 248 dB/m). Another six patients were excluded because of secondary causes of chronic liver disease (hepatitis B, three subjects; C, 2 subjects; autoimmune hepatitis, 1 subject). In total, 331 patients with valid measurements were included in the final analysis: 73 (22.1%) lean patients and 258 (77.9%) obese subjects. The mean age of the whole study group was 56.89 ± 13.07 years; regarding gender, 188 were men, and 143 were women. Hypertension and T2DM were present in 207 (62.5%) and 101 (30.5%) patients, respectively. All other patient characteristics included in our analysis are summarized in Table 1.

### 3.2. Comparison of LSM and CAP Values in Lean- and Obese-NAFLD Patients

The mean value of liver fibrosis for the overall cohort was 7.4 ± 4.2 kPa, with a mean of 6.3 ± 3.3 kPa in the lean group and 7.6 ± 4.3 kPa for the obese patients (*p* = 0.007). The distribution of participants based on LSM values in F0, F1, F2, F3, and F4 was 122 (38.86%), 79 (23.87%), 63 (19.03%), 37 (11.18%), and 30 (9.06%), with the proportion of subjects with at least significant fibrosis (≥F2) being approximately two times higher among obese-NAFLD (43.81%) in comparison with lean-NAFLD patients (23.29%). CAP values were higher according to the subjects’ BMI, with a mean value of 273.3 ± 23.5 dB/m in lean patients and 307.5 ± 41.3 dB/m in the obese subjects (*p* < 0.001). The distribution of lean patients in S1, S2, S3 was 36 (49.32%), 23 (31.51%), and 14 (19.18%). Data regarding the obese subjects and their CAP values showed a significant statistical difference between the two groups regarding liver steatosis, for which patient distribution in S1, S2, and S3 was 54 (20.93%), 39 (15.2%), and 165 (63.95%) (Table 1). In addition, the relative risk for liver fibrosis was considerably higher (OR 2.6, 95% CI 1.5–4.42, *p* < 0.001) in obese individuals, compared with lean subjects. 

### 3.3. Biochemical Profile in Lean versus Obese Subjects

The biochemical profiles of the two groups followed the same patterns. However, according to serological parameters, obese subjects presented a higher level of total cholesterol (*p* = 0.047), LDL-c (*p* = 0.013), SUA (*p* < 0.001), fasting plasma glucose (*p* < 0.001), ALT (*p* = 0.003), AST (*p* = 0.003), GGT (*p* = 0.037), ALP (*p* < 0.001), serum urea (*p* < 0.001), creatinine (*p* = 0.001) and lower HDL-c mean values (*p* < 0.001). Notably, there was no significant statistical difference in the mean value for the triglycerides between the two groups (*p* = 0.437) (Table 1).

### 3.4. Factors Associated with Advanced Fibrosis in Lean and Obese Patients

**Figure 2:**
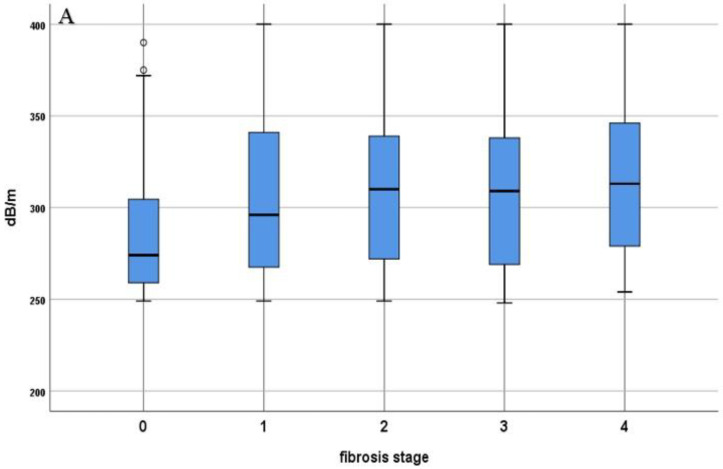

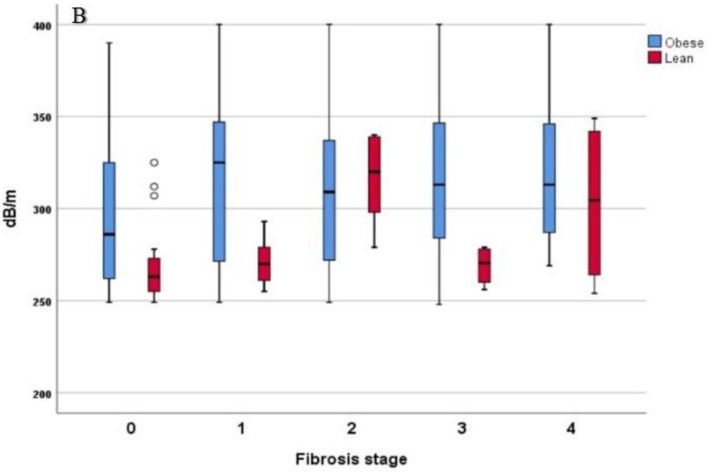
(**A**) Distribution of CAP values according to fibrosis stage; (**B**) distribution of CAP values among lean and obese patients according to fibrosis stage.

Different parameters were found to correlate with the presence of fibrosis, both in lean and obese individuals, such as INR (r = 0.384, *p* < 0.001; r = 0.595, *p* < 0.001), CRP (r = 0.168, *p* = 0.026; r = 0.455, *p* < 0.001), AST (r = 0.370, *p* < 0.001; r = 0.273, *p* = 0.019) and LDL-c (r = 0.164 *p* = 0.008; r = 0.263, *p* = 0.024) (Table 2). Furthermore, there was a strong, positive correlation in the lean group between the presence of fibrosis and total cholesterol (r = 0.508, *p* < 0.001) and triglycerides (r = 0.381, *p* = 0.001). An important feature of our study cohort is the fact that the LSM value was directly proportional with the degrees of liver steatosis (Figure 2).

We conducted univariate, followed by multivariate, linear regression analyses, with the aim to identify the clinical–biochemical parameters associated with advanced liver fibrosis and cirrhosis (Table 3). In the lean group, the multivariate analysis showed that triglycerides (β = 0.256, *p* = 0.013) and INR (β = 0.389, *p* = 0.001) were strongly associated with advanced fibrosis. While AST and CRP did not have a significant value in the multivariate analysis, they were still strongly associated in the univariate analysis, with important degrees of liver fibrosis. Lean-NAFLD with AST higher than normal limit had a higher risk for advanced fibrosis, compared with those with normal levels of liver enzymes (OR 5.3, 95% CI 1.29–21.74, *p* = 0.013). On the other hand, in the obese group, INR (β = 0.235, *p* = 0.039), serum urea (β = 0.123, *p* = 0.048) and ALT (β = 0.182, *p* = 0.042) levels were strongly associated with advanced fibrosis and cirrhosis in the multivariate analysis. Moreover, obese-NAFLD subjects with ALT higher than the normal limit had an increased risk for advanced fibrosis (OR 5.4, 95% CI 2.9–10.29, *p* < 0.001). The presence of both elevated liver aminotransferases in lean-NAFLD individuals had a higher risk for ≥F3 degree (OR 2.22, 95% CI 0.2–23.75, *p* = 0.499), while obese-NAFLD subjects had a more important risk (OR 5.90, 95% CI 3.08–11.28, *p* < 0.001) for advanced liver fibrosis. We also found a significant, negative association between LSM values and platelet count in both lean (β = −0.204, *p* = 0.042) and obese (β = −0.109, *p* =0.031) groups (Table 3). 

## 4. Discussion

In the past few years, it has been generally accepted that NAFLD is no longer a condition to be ignored, with a high risk of progression to end-stage liver disease [26]. As the fatty liver is oftentimes a symptom-free disease, the diagnosis and management are challenging, especially in lean patients, since these individuals do not fit the classic phenotype of obesity. However, they still share a common metabolic profile with obese subjects [27,28,29]. 

In this study, we provided further information regarding the differences between lean and obese patients diagnosed with NAFLD using VCTE with CAP, to better understand and characterize the clinical features and associated risk factors of each group. Despite the fact that both groups followed the same pattern of metabolic changes, the modifications in the obese individuals were more significant than in the lean patients, results similar to those reported by other studies [14,30]. We found that metabolic abnormalities such as a high value of SUA, fasting plasma glucose, CRP, total cholesterol, LDL-c, triglycerides, and low levels of HDL-c were more frequent and more severe in obese subjects. This aspect was reported in the current literature as an intermediate metabolic profile in lean-NAFLD patients, between obese-NAFLD and healthy individuals [30]. Thus, it appears that lean-NAFLD and obese-NAFLD are two distinct entities, with different metabolic and fibrosis profiles and, therefore, with different outcomes in terms of prognosis. In this view, Chahal et al. recently developed a predictive score for NAFLD in lean patients, with good sensibility and specificity, which could be used in screening strategies [31].

The development and progression of NAFLD are known to be strongly associated with the presence of T2DM and hypertension, comorbidities frequently found in the general population. A meta-analysis conducted by Younossi et al. revealed that T2DM was more often found in NAFLD patients, affecting up to 23% of them [32]. In our study, we reported a higher prevalence of T2DM, affecting 101 (30.51%) of patients, 19.18% of them lean, while in the obese group, the prevalence was noticeably higher, at 33.72%. Similar results have been reported by Denkmayr et al., with a prevalence of 17.5% and 45.3% in lean and obese subjects [33]. We also found that hypertension is more frequent in obese patients (68.6% vs. 41.4%, *p* < 0.001) than in lean individuals. Even though the prevalence of hypertension in this group of patients is discordant, the studies in the current literature conclude that this comorbidity is in fact more prevalent in obese patients with NAFLD [33,34,35].

Dyslipidemia, a part of the MeS, is believed to play an important role in the pathophysiology of NAFLD, both in lean and obese patients [36]. The available data are heterogeneous regarding the levels of total cholesterol and triglycerides and the differences between the two groups of patients. Denkmayr et al. reported high mean values of triglycerides and total cholesterol for both lean and obese patients but with no significant statistical difference between the two groups [33]. On the other hand, a study conducted by Khayyat showed a higher mean value of total cholesterol and lower values of triglycerides among lean individuals [37]. In a systematic review and meta-analysis by Young et al., the cholesterol and triglycerides levels were higher in obese patients with NAFLD, compared with lean individuals [38]. Following this trend, the patients in our study presented consistent high levels of triglycerides and total cholesterol. Further analysis showed a significant statistical difference between the two groups regarding the total cholesterol (*p* = 0.047), HDL-c (*p* < 0.001) and LDL-c (*p* = 0.013) values but with no major difference concerning the levels of triglycerides (*p* = 0.437). Thus, lean and obese patients have metabolic similarities in what concerns triglycerides, sharing common risk factors for developing hepatic fat accumulation. 

With regard to the levels of liver enzymes, there were significant differences between the lean and obese patients with NAFLD, with mean AST and ALT values of 28.1 ± 14.3, 31.9 ± 20.5 and 35.6 ± 20.3 (*p* = 0.003), 46.2 ± 27.7 (*p* = 0.003) kPa, respectively. A strong correlation was noted between the presence of fibrosis and increased AST levels in lean subjects (r = 0.27, *p* = 0.019), whereas the obese individuals presented a strong correlation between both AST (r = 0.37, *p* < 0.001) and ALT (r = 0.34, *p* < 0.001) and the presence of liver fibrosis. These results were in accordance with other studies, which reported higher mean values of liver enzymes in the obese-NAFLD patients [5,14,33,39]. On the other hand, as previously presented, the result up to this point are heterogeneous, with other studies showing mean values of AST and ALT higher in lean-NAFLD patients [40,41]. Liver enzymes are indeed usually increased in patients with NAFLD, but not all subjects have these modifications. Burger et al. reported that higher ALT levels were correlated with hepatic fat accumulation and insulin resistance, results also advocated by Francazani et al. [42,43]. However, a recent study conducted by Ulasoglu et al. among NAFLD biopsy-proven patients concluded that despite the similar prevalence of advanced liver fibrosis among subjects with or without normal liver enzymes, those with hepatocytolysis had more severe histological liver findings [44]. Thus, our results highlight the risk of rapid progression to liver cirrhosis also among lean individuals, mostly an apparently clinically healthy population, due to liver necrotic inflammatory activity in those with elevated liver enzymes. A more careful screening of NAFLD in the lean population, not without necessarily relying on phenotypic criteria, is required. 

In this study, most of the patients had no liver fibrosis, but there was an important number of them with advanced fibrosis (F3: 37, 11.18%) and liver cirrhosis (F4: 30, 9.06%). A noteworthy finding was the fact that the LSM value was directly proportional to the degree of liver steatosis. We also found by univariate analysis a significant positive association between CRP (β = 0.256, *p* = 0.029), AST (β = 0.310, *p* = 0.008), triglycerides (β = 0.268, *p* = 0.022), and advanced liver fibrosis and cirrhosis in the lean group. In the multivariate analysis, platelet count was negatively associated with F3 and F4 (β = −0.204, *p* = 0.042), while triglycerides presented a positive association (β = 0.256, *p* = 0.013) for patients with a BMI <25 kg/m². Regarding the obese group, the multivariate regression analysis showed a positive correlation of advanced liver fibrosis with serum urea levels and the presence of hypertension and ALT values, while the platelet count was negatively correlated (β = −0.178, *p* = 0.031), results that are in accordance with those from the current literature [45,46]. On the other hand, in a study by Verma et al., with biopsy-proven NAFLD, more than 30% of patients with normal ALT levels showed evidence of advanced fibrosis; therefore, the important risk of liver injury should be suspected even in patients with normal ALT levels [47]. In our cohort, the risk for advanced liver fibrosis was higher among obese patients with high ALT values, while in the lean group, a more important risk for advanced fibrosis was found in individuals with high AST values. Natarajan et al. showed similar data, concluding that patients with persistently elevated ALT levels and hepatic steatosis are at high risk for developing advanced chronic liver disease and, consequently, HCC [48].

According to the European Association for the Study of the Liver NAFLD guidelines, screening for NAFLD/NASH is not recommended in the general population, unless there are patients at high risk to develop this disease. However, we observed an important prevalence of advanced (F3 and F4) liver fibrosis (13.7%) among lean patients, which is why a screening program should be considered, even in apparently healthy subjects with metabolic changes, regardless of the severity of the modification. 

In our study, the main limitation was the absence of LB in the diagnosis of NAFLD, as it is appropriate only for patients with a high risk of NASH. The absence of the overweight group could be considered another limitation. However, this brings more relevant data regarding the importance of obesity in the development of NAFLD; in addition, by disregarding the “middle/grey” group, we emphasized the metabolic similarities in the analyzed patients. Another limitation was the absence of BF% evaluation by MRI, CT scans, or DXA. Even if these methods have been validated, they have important drawbacks such as the lack of accessibility for the sole purpose of BF% assessment, being time-consuming, radiation exposure, lack of cost efficiency, and manual image analysis, which can interfere with daily clinical practice. Despite these limitations, our study brings considerable results with respect to liver fibrosis in lean and obese patients with NAFLD. Firstly, a strength of our study was that all included subjects were evaluated using VCTE with CAP for the diagnosis and staging of liver fibrosis and steatosis. This is a quantitative method that provides accurate data regarding fat accumulation and fibrosis degree, leading to a low rate of bias regarding underdiagnosed liver steatosis in our cohort. Secondly, the prospective design of this study including carefully selected patients based on strict criteria highlights the risk of advanced liver fibrosis both in lean and obese subjects. Further studies are needed for establishing the resemblances between these two groups, dichotomizing between metabolically healthy and unhealthy populations regardless of the clinical appearance.

## 5. Conclusions

NAFLD is most likely a multicausal disease and its development and progression are yet to be fully understood. It is commonly associated with obese individuals, but lean-NAFLD patients are no longer an extraordinary situation, having an important risk of developing advanced hepatic fibrosis, cirrhosis, and hepatocellular carcinoma. However, patients with lean-NAFLD usually have a milder clinical and biochemical phenotype, with lower values at the LSM evaluation when compared with obese subjects. 

## Figures and Tables

**Table 1:** Baseline characteristics of patients.

	Overall Cohort	Lean	Obese	*p*-Value
Patients characteristics	*n*= 331	*n*, (%) = 73 (22.1)	*n*, (%) = 258 (77.9)	
Age (years)	56 ± 13	52 ± 15	58 ± 11	0.001
Height (cm)	170 ± 9	172 ± 8	170 ± 9	0.076
Weight (kg)	87 ± 15	68 ± 8	93 ± 12	<0.001
Gender female	143 (43.20%)	36 (49.32%)	107 (41.47%)	0.232
Gender male	188 (56.80%)	37 (50.68%)	151 (58.53%)	0.158
BMI (kg/m²)	30 ± 4	22 ± 1	32 ± 2	<0.001
LSM (kPa)	7.40 ± 4.22	6.37± 3.36	7.69 ± 4.39	0.007
CAP (dB/m)	299 ± 40	273 ± 23	307 ± 41	<0.001
Platelet count (10^9^/L)	246 ± 74	238 ± 65	248 ± 76	0.271
INR	1.02 ± 0.16	1.01 ± 0.17	1.03 ± 0.16	0.532
CRP (mg/dL)	0.52 ± 1.50	0.38 ± 0.53	0.56 ± 1.68	0.154
Fasting plasma glucose (mg/dL)	110 ± 38	96 ± 20	114 ± 41	<0.001
Serum urea (mg/dL)	38 ± 10	33 ± 7	39 ± 10	<0.001
Creatinine (mg/dL)	0.8 ± 0.1	0.8 ± 0.1	0.8 ± 0.1	0.001
ALT (IU/L)	43 ± 26	31 ± 20	46 ± 27	0.003
AST (IU/L)	33 ± 19	28 ± 14	35 ± 20	0.003
GGT (IU/L)	44 ± 37	37 ± 31	46 ± 38	0.037
ALP (IU/L)	85 ± 31	73 ± 21	89 ± 33	<0.001
Total bilirubin (mg/dL)	0.7 ± 0.3	0.7 ± 0.2	0.7 ± 0.3	0.701
Total cholesterol (mg/dL)	215 ± 39	207 ± 38	217 ± 40	0.047
Triglycerides (mg/dL)	157 ± 71	153 ± 51	159 ± 75	0.437
Albumin (g/dL)	4.6 ± 2	4.5 ± 0.4	4.7 ± 2.3	0.297
LDL-c (mg/dL)	128 ± 39	117 ± 41	131 ± 38	0.013
HDL-c (mg/dL)	45.13 ±11	49.41 ± 10	43 ± 11	<0.001
Serum uric acid (mg/dL)	4.9 ± 1.4	4.2 ± 1.3	5.1 ± 1.4	<0.001
Fibrosis				
F0, *n* (%)	122 (38.86%)	40 (54.79%)	82 (31.78%)	<0.001
F1, *n* (%)	79 (23.87%)	16 (21.92%)	63 (24.42%)	0.658
F2, *n* (%)	63 (19.03%)	7 (9.59%)	56 (21.71%)	0.020
F3, *n* (%)	37 (11.18%)	6 (8.22%)	31 (12.02%)	0.363
F4, *n* (%)	30 (9.06%)	4 (5.48%)	26 (10.08%)	0.227
Steatosis				
S1, *n* (%)	90 (27.19%)	36 (49.32%)	54 (20.93%)	<0.001
S2, *n* (%)	62 (18.73%)	23 (31.51%)	39 (15.12%)	0.002
S3, *n* (%)	179 (54.08%)	14 (19.18%)	165 (63.95%)	<0.001
Hypertension, *n* (%)	207 (62.54%)	30 (41.10%)	177 (68.60%)	<0.001
T2DM, *n* (%)	101 (30.51%)	14 (19.18%)	87 (33.72%)	0.017

BMI, body mass index; ALT, alanine aminotransferase; AST, aspartate aminotransferase; GGT, gamma-glutamyl transferase; ALP, alkaline phosphatase; LDL-c, low-density lipoprotein cholesterol; HDL-c, high-density lipoprotein cholesterol; LSM, liver stiffness measurement; CAP, controlled attenuation parameter; CRP, c-reactive protein; INR, international normalized ratio.

**Table 2:** Parameters associated with the presence of fibrosis.

Parameter	Obese		Lean	
	*r*	*p* Value	*r*	*p* Value
Age	0.043	0.492	0.130	0.273
Height	0.140	0.024	−0.107	0.369
Weight	0.178	0.004	−0.103	0.388
BMI	0.100	0.109	−0.034	0.776
Platelet count (G/L)	−0.287	<0.001	−0.034	0.776
INR	0.384	<0.001	0.595	<0.001
CRP (mg/dL)	0.168	0.026	0.455	<0.001
Fasting plasma glucose (mg/dL)	0.157	0.011	−0.003	0.979
Serum urea (mg/dL)	0.120	0.054	−0.128	0.281
Creatinine (mg/dL)	0.104	0.095	0.162	0.172
ALT (IU/L)	0.348	<0.001	0.015	0.900
AST (IU/L)	0.370	<0.001	0.273	0.019
GGT (IU/L)	0.426	<0.001	0.109	0.361
ALP (IU/L)	0.238	<0.001	0.055	0.644
Total bilirubin (mg/dL)	0.240	<0.001	0.106	0.370
Total cholesterol (mg/dL)	0.120	0.054	0.508	<0.001
Tryglicerides (mg/dL)	0.007	0.910	0.381	0.001
Albumin (g/dL)	0.082	0.193	−0.294	0.012
LDL-c (mg/dL)	0.164	0.008	0.263	0.024
HDL-c (mg/dL)	−0.136	0.028	−0.016	0.896
Serum uric acid (mg/dL)	0.216	<0.001	0.134	0.257

BMI, body mass index; ALT, alanine aminotransferase; AST, aspartate aminotransferase; GGT, gamma-glutamyl transferase; ALP, alkaline phosphatase; LDL-c, low-density lipoprotein cholesterol; HDL-c, high-density lipoprotein cholesterol; CRP, c-reactive protein; INR, international normalized ratio.

**Table 3:** Factors associated with advanced liver fibrosis using univariate and multivariate linear regression analyses.

	Lean				Obese			
Variable	Univariate		Multivariate		Univariate		Multivariate	
	β	*p*	β	*p*	β	*p*	β	*p*
Age	0.126	0.287			−0.045	0.473		
Gender	0.085	0.474			−0.164	0.008	−0.014	0.878
Height	−0.101	0.393			0.214	0.001	0.173	0.150
Weight	−0.055	0.643			0.196	0.002	0.039	0.690
BMI	0.031	0.796			0.029	0.640		
Platelet count (G/L)	−0.372	0.001	−0.204	0.042	−0.230	<0.001	−0.109	0.031
INR	0.518	<0.001	0.389	0.001	0.338	0.001	0.235	0.039
CRP (mg/dL)	0.256	0.029	−0.025	0.813	0.008	0.900		
Fasting plasma glucose (mg/dL)	−0.139	0.239			0.050	0.424		
Serum urea (mg/dL)	−0.023	0.848			0.148	0.018	0.123	0.048
Creatinine (mg/dL)	0.132	0.265			0.072	0.248		
ALT (IU/L)	0.106	0.373			0.283	<0.001	0.182	0.042
AST (IU/L)	0.310	0.008	0.135	0.203	0.281	<0.001	−0.078	0.518
GGT (IU/L)	−0.026	0.828			0.375	<0.001	0.148	0.054
ALP (IU/L)	0.044	0.711			0.224	<0.001	0.024	0.729
Total bilirubin (mg/dL)	0.159	0.180			0.069	0.273		
Total cholesterol (mg/dL)	0.264	0.024			0.135	0.030	−0.055	0.419
Triglycerides (mg/dL)	0.268	0.022	0.256	0.013	0.026	0.677		
Albumin (g/dL)	−0.222	0.059			−0.075	0.228		
LDL-c (mg/dL)	0.073	0.541			0.151	0.015		
HDL-c (mg/dL)	0.094	0.429			−0.133	0.032	0.019	0.774
Serum uric acid (mg/dL)	0.043	0.719			0.240	<0.001	0.095	0.183
Hypertension	0.153	0.196			0.199	0.001	0.133	0.025
T2DM	−0.093	0.434			0.055	0.380		

BMI, body mass index; ALT, alanine aminotransferase; AST, aspartate aminotransferase; GGT, gamma-glutamyl transferase; ALP, alkaline phosphatase; LDL-c, low-density lipoprotein cholesterol; HDL-c, high-density lipoprotein cholesterol; CRP, c-reactive protein; INR, international normalized ratio.

## Data Availability

The data presented in this study are available on request from the corresponding author. The data are not publicly available because they are the property of the Institute of Gastroenterology and Hepatology, Iasi, Romania.
